# Mongolian and Japanese Joint Conference on "Echinococcosis: diagnosis, treatment and prevention in Mongolia" June 4, 2009

**DOI:** 10.1186/1756-3305-3-8

**Published:** 2010-02-08

**Authors:** A Gurbadam, D Nyamkhuu, G Nyamkhuu, A Tsendjav, O Sergelen, B Narantuya, Z Batsukh, G Battsetseg, B Oyun-Erdene, B Uranchimeg, D Otgonbaatar, D Temuulen, E Bayarmaa, D Abmed, S Tsogtsaikhan, A Usukhbayar, K Smirmaul, J Gereltuya, A Ito

**Affiliations:** 1 Health Science University of Mongolia (HSUM), Ulaanbaatar, Mongolia; 2 National Center of Communicable Diseases (NCCD), Ministry of Health, Ulaanbaatar, Mongolia; 3 State Central Clinical Hospital (SCCH), Ulaanbaatar, Mongolia; 4 State Research Center on Maternal and Child Health (SRCMCH), Ulaanbaatar, Mongolia; 5 Institute of Veterinary Medicine (IVM), Ulaanbaatar, Mongolia; 6 Pathology Center (PC), Ministry of Health, Ulaanbaatar, Mongolia; 7 Center of Infectious Diseases with Natural Foci (CIDNF), Ministry of Health, Ulaanbaatar, Mongolia; 8 Mongolia V.E.T NET, Ulaanbaatar, Mongolia; 9 Asahikawa Medical College (AMC), Asahikawa, Japan

## Abstract

The first Mongolian-Japanese Joint Conference on "Echinococcosis: diagnosis, treatment and prevention in Mongolia" was held in Ulaanbaatar on June 4^th^, 2009. It was the first chance for Mongolian experts (clinicians, pathologists, parasitologists, biologists, epidemiologists, veterinarians and others working on echinococcosis) joined together. Increase in the number of cystic echinococcosis (CE) cases year by year was stressed. CE in children may be more than adult cases. Alveolar echinococcosis was suspected chronic malignant hepatic tumors or abscesses. Main discussion was as to how to introduce modern diagnostic tools for pre-surgical diagnosis, how to establish the national system for the data base of echinococcosis with the establishment of a network system by experts from different areas. The importance of molecular identification of the parasites in domestic and wild animals was also stressed.

## Report

D Nyamkhuu, D Otgonbaatar, G Nyamkhuu, O Sergelen, A Gurbadam, Z Batsukh, K Smirmaul and A Ito had often been discussing on how to establish the reference center on echinococcosis in Mongolia from 2004. Ito visited Ulaanbaatar for the first time in May 1995 as one of the parasitology delegates from the United States with Cross from the Uniformed Services University of Health Sciences in Bethesda, Maryland. At that time, Goosh, Head of the Department of Surgery at the Mongolian National Medical University (= Health Sciences University of Mongolia (HSUM) from 2003) explained the situation of echinococcosis in Mongolia. He concluded that most cases of echinococcosis in Mongolia were cystic echinococcosis (CE), since the life style of Mongolians was nomadic. Only 5 alveolar echinococcosis (AE) cases were confirmed before 1995. However, there is no published record, mainly due to the collapse of the Soviet Union. Clinical cases of echinococcosis in Mongolia were managed by surgeons. In 1950, 7.8% of all surgical patients in Mongolia were CE, whereas it was 1.9% in 1990 [1]. Also, Davaatseren and others reported that CE was the cause for 18% of the surgical cases in the First Hospital of Ulaanbaatar (i.e. SCCH) in 1993 [2,3].

After the Soviet Union collapsed in 1990, control programs for deworming dogs in Russia and former Soviet Union controlled states were also affected. Therefore, a resurgence of CE through the increase in number of dogs infected with *E. granulosus *was expected to become a serious public health risk for the people in these areas including Mongolia [3,4].

On June 4, 2009, Gurbadam and Temuulen at the Department of Medical Biology and Histology, HSUM, set up the first Mongolian-Japanese Joint Conference on "Echinococcosis: diagnosis, treatment and prevention in Mongolia" at HSUM when Ito visited Ulaanbaatar. It was the first chance for 19 Mongolian experts involved in echinococcosis to join together. There has been no other such meeting where clinicians, pathologists, parasitologists, biologists, epidemiologists, veterinarians and others working on echinococcosis in Mongolia joined together. Surgeons working in HSUM, SCCH, SRCMCH, pathologists from CP, HSUM, SCCH, immunologists, biologists, parasitologists from HSUM, and veterinarians from IVM and V.E.T. Net Mongolia NGO and a medical official, Luo from WHO/WPRO Ulaanbaatar as an observer attended.

The meeting was chaired by Gurbadam and opened with welcome remarks by the Dean, Batbaatar, School of Biomedicine, HSUM. Nyamkhuu, Director General of NCCD explained the historical background on the action plan towards the establishment of a national reference center for control of echinococcosis in Mongolia.

Two speakers gave special lectures. The first speaker was Ito. His topics were the "Recent advances in serodiagnosis on echinococcosis" and "Discussion on the new bilateral proposal on the establishment of centers for control of echinococcosis in Mongolia". He briefly explained three main topics. The first was the life cycles of *Echinococcus granulosus *and *E. multilocularis *along with their geographic distribution in the world [5]. The second was the recent advances in serodiagnosis of AE using recombinant Em18 with data from international joint projects with German [6], French and Swiss groups (unpublished) and Japanese [7]. The third topic was the similar approach using recombinant AgB8/1 for CE. The importance of the combination of abdominal imaging and serology was stressed according to recommendations from WHO (2001) [8]. The representative stressed the importance of a depository of the case records in Mongolia. Then, Naymkhuu from SCCH explained the most recent 5 AE cases in Mongolia (1982, 2002, 2006, 2007, 2009) [9]. Pre-surgical diagnoses for all AE cases were suspected chronic malignant hepatic tumors or abscesses. There was no application of serology for pre-surgical diagnosis. Ito showed antibody responses in AE case 4 (2007) and stressed that he expected strong positive responses in case 5 (2009) when he could check it after the meeting. [It has become evident that case 5 shows very strong antibody responses to RecEm18, the most sensitive and specific marker for the presence of active AE lesions [6,7,9,10]. Furthermore, haplotype network analysis of the most recent 3 histopathological specimens has revealed the presence of the two, Asian and Inner Mongolian genotypes reported by Nakao and others [5] in Mongolia [10]. Therefore, it is a very interesting area on the geographic distribution of the two genotypes of *E. multilocularis *in Mongolia and may be also in Russia and China. Also, there are many domestic and wild animals which may become intermediate hosts for *E. granulosus sensu lato *[11]. Therefore, molecular approaches on *Echinococcus *spp. and their host spectra will become interesting and important for genetic diversity, geographic distribution and co-evolution of *Echinococcus *spp. in Mongolia, Russia and China [5].

The second speaker was Tsendjav from SRCMCH. He summarized 25 pediatric CE cases from 2008 (19 in 2008, 6 from Jan until May 2009; 15 boys and 10 girls including 4 children under the age of 5-years) at SRCMCH. All participants stressed that the number of CE cases had been increasing year by year.

Through open discussion, Narantuya from SCCH summarized a total of 144 (63 males and 81 females) CE cases (1989-2009 June). In 2008 and 2009 until June, 13 and 11 cases, respectively, were surgically confirmed to be CE at SCCH. There is a tendency to see more CE cases year by year. Among them, 98 cases had liver cysts (68.1%). Sergelen from HSUM explained 9 complicated CE cases with surgery out of UB in 2008. Therefore, we had at least 22 CE cases in 2008 in Mongolia. So, pediatric CE cases in UB in 2008 were more than adult CE cases in UB. It suggests that adult cases are more asymptomatic than pediatric cases. However, we in Mongolia have had no idea how to record the cases and how to educate people especially school children how dogs are high risk for transmission of this disease. We all discussed how to keep the database and share among the experts in Mongolia systematically. NCCD will establish the reference center for human cases with closer contact with Word Health Organization (WHO/WPRO) in Ulaanbaatar, whereas IVM will report animal cases and notify the Food and Agricultural Organization of the United Nations (FAO) in Ulaanbaatar. As echinococcosis is a zoonosis, periodical exchange of mutual information will become a great benefit for launching the active strategy for control and prevention of echinococcosis in Mongolia. The specimens from humans and animals may be deposited at CIDNF. Pediatric cases should be reported not only to Ministry of Health, Mongolia but also to the United Nations Children's Fund (UNICEF) in Ulaanbaatar in order to save children's lives.

Although this meeting was a bilateral meeting on echinococcosis, similar approaches for discussion on the strategy for future control of echinococcosis will become important especially in countries in former Soviet Union, Russia and China [4,12-14].
